# ROS and COVID

**DOI:** 10.3390/antiox11020339

**Published:** 2022-02-09

**Authors:** Periklis Vardakas, Zoi Skaperda, Fotios Tekos, Demetrios Kouretas

**Affiliations:** Department of Biochemistry and Biotechnology, University of Thessaly, Viopolis, Mezourlo, 41500 Larissa, Greece; periklis_vardakas94@hotmail.com (P.V.); zoskaper@bio.uth.gr (Z.S.); ftekos@uth.gr (F.T.)

In recent years, the pandemic of coronavirus disease 19 (COVID-19), an infectious disease caused by variants of severe acute respiratory syndrome coronavirus 2 (SARS-CoV-2), has indisputably emerged as the predominant public health issue. In view of the fact that the disease severity and risk of mortality exhibit significant interindividual variability, the identification of the contributing factors poses a major challenge to the scientific community and might be of outmost importance for the disease control and/or treatment. At a molecular level, a growing body of evidence indicates a close connection between specific biological responses, i.e., inflammation and oxidative stress, and COVID-19 pathogenesis and progression to severe clinical phenotypes that could potentially be fatal. In broad terms, the inflammatory responses stimulated by SARS-CoV-2 infection disrupt redox homeostasis and induce elevated levels of oxidative stress that, in turn, maintain the inflammatory state, thus generating a vicious cycle. Hence, the elucidation of the molecular mechanisms implicated in the disease pathophysiology could play a decisive role in COVID-19 understanding and presumably contribute to the development of novel therapeutic strategies for patients infected with SARS-CoV-2.

Based on the above, in this Special Issue of *Antioxidants*, “ROS and COVID”, 10 interesting articles investigate the relationship between COVID-19 and reactive oxygen species (ROS), molecular entities deeply involved in the disturbance of redox equilibrium and the incitement of inflammatory responses. At first, five articles, authored by Duca et al. [1], Žarković et al. [2], Xu et al. [3], Silvagno et al. [4], and Tekos et al. [5], emphasize the crucial role of redox milieu in disease aggravation, and demonstrate clear evidence of redox imbalance and active inflammation in severe COVID-19 phenotypes. Then, four articles, authored by Martínez [6], Klouda and Stone [7], Fusco et al. [8], and Abbasi et al. [9], discuss the potential efficacy of specific pharmaceuticals in COVID-19 treatment, on the basis of their antioxidant capacity, also describing a non-invasive therapeutic approach that might improve the survival rate of critically ill patients. Finally, Clavo et al. [10] examine the putative value of ROS-oxidizing properties in the decontamination of SARS-CoV-2 from personal protective equipment (PPE).

Recent studies have demonstrated a strong interrelation between host redox status and COVID-19 severity; for that reason, the pathophysiological conditions associated with redox dysregulation constitute risk factors for severe outcomes in patients infected with SARS-CoV-2. Hypoxic patients previously diagnosed with acute respiratory distress syndrome (ARDS), chronic obstructive pulmonary disease (COPD) or COVID-19 exhibit perturbations of blood redox and iron homeostasis depending on the clinical condition [1]. More specifically, in patients experiencing acute hypoxia, i.e., ARDS and COVID-19 patients, the assessment of biomarkers reflecting blood antioxidant capacity and iron regulation indicates the manifestation of adverse effects, whereas in the case of COPD patients, chronic hypoxia causes the activation of blood adaptive mechanisms. Additionally, early findings from a pilot study involving critically ill COVID-19 patients reveal disturbances in blood redox equilibrium, characterized by extremely low levels of total antioxidant capacity and advanced levels of lipid peroxidation products [2]. Interestingly, lung accumulation of 4-hydroxynonenal (HNE)–protein adducts, detected at high levels in deceased patients, is an aggravating factor for disease outcome. Furthermore, the redox state of nicotinamide adenine dinucleotide (NAD(H)) and the genetic background of surfactant protein A (SP-A) might play a pivotal role in the functions of alveolar macrophages and, therefore, in innate immunity [3]. Exposure of male and female SP-A2 and SP-A knockout (KO) mice to ozone, a powerful oxidizing agent, for 3 h promotes the oxidation of NAD(H) of alveolar macrophages in KO mice, whereas in SP-A2 mice, stimulates the generation of higher levels of mitochondrial ROS and causes a greater oxidative shift of NAD(H) in males. Moreover, the evaluation of NAD(H) redox state in female mice denotes that the genetically modified mice lacking SP-A are particularly susceptible to oxidation. Comprehensively, under oxidizing conditions, the redox state of NAD(H) exhibits a sex-dependent response, in which SP-A2 is a major contributor. The intracellular levels of the reduced form of glutathione (GSH) are potent modulators of redox homeostasis, exerting significant cytoprotective activities, among which the direct and indirect ROS neutralization [4]. Considering that the depletion of GSH pools is a common phenomenon in clinical conditions related to a higher risk of severe COVID-19, the enhancement of the specific antioxidant system could be examined as a potential strategy for the prevention and/or treatment of serious illness. Within this context, dietary antioxidant supplementation, the adoption of modern dietary patterns that promote metabolic health, e.g., intermittent fasting, and regular physical activity might be key determinants for an improvement in the antioxidant defense mechanisms and innate immunity of a host, thus preventing severe COVID-19 infection and complications [5].

In efforts to develop or repurpose drugs for the treatment of COVID-19, an evaluation of antioxidant properties in putative therapeutic agents could provide valuable insights into their effectiveness. With this in mind, a theoretical investigation designed to examine the free-radical-scavenging capacity of promising drugs for COVID-19 treatment stresses that their ability to donate or accept electrons may also be a double-edged sword [6]. In terms of their electron donor–acceptor properties, elbasvir, an antiviral drug used to treat hepatitis C, and valrubicin, an antiviral drug, are the strongest electron donor and electron acceptor, respectively. Nevertheless, electron-accepting molecules, such as valrubicin, may also oxidize endogenous biomolecules and, therefore, cause disturbances in redox equilibrium. On the contrary, molnupiravir and ivermectin, antiviral drugs with potent electron-donating capabilities, are weak electron acceptors, rendering them promising drugs for COVID-19 treatment. Furthermore, chloroquine (CQ) and hydroxychloroquine (HCQ), drugs originally developed as antimalarials, have been repurposed for the treatment of COVID-19, however, several concerns regarding their safety and effectiveness have been raised [7]. Particularly, the administration of CQ/HCQ in patients infected with SARS-CoV-2 may induce systemic oxidative stress, promote lipid peroxidation, change the phenotype of macrophages, affect immune responses, and cause damage to type II alveolar cells. In contrast, adelmidrol, a compound exerting strong antioxidant and anti-inflammatory activities, is a promising therapeutic agent for fibrotic lung diseases, that may emerge as severe post-COVID-19 complications [8]. Specifically, a 21-day course of adelmidrol (10 mg/kg) administration in mice suffering from bleomycin-induced pulmonary fibrosis attenuates lung inflammatory responses by modulating the overexpression of pro-inflammatory cytokines and ameliorates oxidative stress by reducing the generation of free radicals, restoring antioxidant defense mechanisms, and decreasing lipid peroxidation. Additionally, lung histopathological findings indicate that adelmidrol administration prevents severe lung injury and, overall, improves the survival rate of mice with pulmonary fibrosis. Finally, an intriguing finding derived from a study investigating the impact of SP-A genetics on survival of aged mice affected by bacterial pneumoniae brings to the forefront a non-invasive ventilatory strategy with potential applications in COVID-19 treatment [9]. To be more specific, exposing patients infected with SARS-CoV-2 to a high flow of heated and humidified filtered air may improve the protective functions of the respiratory epithelium, increase alveolar recruitment, enhance oxygenation, and decrease breathing effort. 

Ozone, a ROS member and a strong oxidant, as previously stated, exhibits potent virucidal activities through oxidative stress which, under specific conditions, could contribute to the decontamination of PPE from SARS-CoV-2 [10]. Specifically, exposure of medical gowns and face masks contaminated with heat-inactivated SARS-CoV-2 to ozone, at concentrations ranging from 4000 to 40,000 ppm, efficiently eliminates the virus, in most cases, within a few seconds. Notably, under extremely high levels of relative humidity, i.e., 99%, ozone can eliminate the virus from the surface of gowns at lower concentrations, ranging from 4 to 6.5 ppm, but requires longer exposure time intervals. 

In summary, the articles included in the present Special Issue approach the global health crisis of COVID-19 from the perspective of redox biology, focusing on the molecular interplay between ROS and disease pathogenesis and progression. Viral infection stimulates the generation of elevated levels of ROS that disrupt redox homeostasis and elicit oxidative stress and inflammation, biological responses closely related to disease aggravation. Therefore, strategies aiming to enhance the host redox status may provide solid protection against serious illness, whereas in the case of severe COVID-19, the administration of therapeutic agents that exert potent antioxidant activities may reduce the risk of mortality. 
